# Distinct Effects of Acute Aerobic Exercise on Declarative Memory and Procedural Memory Formation

**DOI:** 10.3390/brainsci10100691

**Published:** 2020-09-30

**Authors:** Xuru Wang, Rui Zhu, Chenglin Zhou, Yifan Chen

**Affiliations:** School of Psychology, Shanghai University of Sport, Shanghai 200438, China; 17858950731@163.com (X.W.); lohasmo@163.com (R.Z.); zhouchenglin@sus.edu.cn (C.Z.)

**Keywords:** acute aerobic exercise, declarative memory, procedural memory, coding period, consolidation period

## Abstract

*Objective:* To investigate the different effects of acute aerobic exercise on the formation of long-term declarative memory (DM) and procedural memory (PM). *Methods:* Twenty-two young men completed DM and PM tasks under three experimental conditions: pre-acquisition exercise, post-acquisition exercise, and no exercise (control). The DM task encompassed word learning, free recall tests both immediately and 1 h later, and a recognition test conducted 24 h after word learning. A serial reaction time task (SRTT) was utilized to assess exercise effects on PM. The SRTT included a sequence learning phase followed by sequence tests 1 h and 24 h later. The exercise program consisted of 30 min of moderate-intensity aerobic exercise. *Results:* In the DM task, compared to the control condition, pre-acquisition exercise, but not post-acquisition exercise, enhanced free recall performance significantly 1 h and 24 h later. The target word recognition rate and discriminative index (d′) of the recognition test were significantly enhanced in both exercise conditions compared to the control condition. In the PM task, we observed significantly reduced (improved) reaction times at the 24-h test in the post-acquisition exercise condition compared to in the control condition. *Conclusion:* Acute aerobic exercise may enhance long-term DM and PM via effects on different processing periods. For DM, exercise had a pronounced effect during the encoding period, whereas for PM, exercise was found to have an enhancing effect during the consolidation period.

## 1. Introduction

In recent years, there has been a growing interest in understanding the effects of exercise on cognitive functions. Although much of the literature examining this relationship has focused on chronic exercise paradigms, acute aerobic exercise effects on cognitive functions have been reported [1,2], especially effects on memory [3,4]. A single exercise bout has been shown to induce plasticity-promoting effects on both a molecular and a systems level, and to lead to increases in arousal [2] and cerebral blood flow [5]. Exercise-induced changes in brain structures [6] may potentially facilitate memory and learning processes [7].

Memory encompasses the faculties by which the brain encodes, stores, and retrieves information. Regarding storage duration, information may be held quite briefly, as is the case in sensory processing or may be stored in short-term, intermediate-term, or long-term memory [8]. Importantly, there are distinct types of memory, wherein the information processing system may function explicitly, as in declarative memory (DM), or implicitly, as in procedural memory (PM) [9]. The conscious, intentional recollection of factual information, previous experiences, and concepts is considered to be DM [10]. Generally, DM is established through gradual learning, such as learning through multiple presentations of a stimulus and response. DM can be divided into two categories: episodic memory, which stores specific personal experiences, and semantic memory, which stores factual information [11]. Conversely, PM supports task performance without conscious awareness of one’s previous experiences with the task. PM is developed through procedural learning, which is essential for the development of any motor skill or cognitive activity.

Because early explorations of aerobic exercise effects on memory did not distinguish between short- and long-term memory, it was unclear whether aerobic exercise can facilitate long-term memory. Meanwhile, some evidence indicated that acute aerobic exercise failed to improve short-term memory performance, whereas it played a positive role in facilitating long-term memory [3]. In more recent studies, positive impacts of acute aerobic exercise on long-term memory, including DM [12,13] and PM [14,15], have been reported. Moreover, these positive effects have been related to molecular processes during the encoding and consolidation stages of long-term memory [16]. Although acute aerobic exercise has been shown to facilitate long-term memory significantly, it is not yet clear which memory-formation processing period benefits from this facilitative effect.

It is possible that the effects of acute aerobic exercise on different processing periods of long-term memory formation may differ between types of memory. Studies using DM materials (such as stories) have suggested that acute aerobic exercise improved long-term memory performance primarily via a positive influence on encoding [4,17]. Meanwhile, positive effects of acute aerobic exercise on motor sequence learning have been linked mainly to enhanced consolidation [18]. Notably, differing intensity exercise paradigms may differentially affect temporally distinct forms of memory [19,20]. Specifically, it has been suggested that high-intensity exercise performed shortly after memory encoding may not affect long-term memory function [6], whereas moderate-intensity exercise has been widely reported to enhance multiple stages of memory formation [19]. Therefore, because the sorts of memory materials used have been variable across studies and because there is a potential interference caused by different exercise intensities, it has been difficult to make direct comparisons between DM and PM findings. The processing mechanisms by which acute aerobic exercise influences particular types of memory remain to be further explored.

The aim of the present study was to analyze the effects of acute aerobic exercise on DM and PM using the same acute aerobic exercise program, with a consistent intensity, across both settings. Based on previous studies [3,4], a 30-min moderate-intensity acute aerobic exercise program was employed in the present study design. DM was assessed via a word learning task with free recall and recognition tests. PM was assessed with a serial reaction time task (SRTT). Based on previous findings in the literature, we hypothesized that acute aerobic exercise would facilitate the encoding phase of DM recall and would facilitate the consolidation phase of PM.

## 2. Materials and Methods

### 2.1. Participants

Twenty-two right-handed students (22 males, mean age = 21.6 ± 3.0; mean body mass index = 22.4 ± 2.1) without intensive athletic experience were recruited randomly from our university. The sample size met the criteria of a power analysis assuming a 3 by 3 repeated-measures design, an alpha of 0.05, a power of 0.9, and an effect size of 0.25. Participants completed the Physical Activity Readiness Questionnaire to determine if it was safe for them to exercise. They also completed the International Physical Activity Questionnaire (Chinese version) to confirm that each had a metabolism of ≥600 metabolic equivalents. The study followed the ethical guidelines of the Declaration of Helsinki and was approved by the Research Ethics Committee at Shanghai University of Sport (approval number 2015003).

### 2.2. Task and Procedures

#### 2.2.1. Acute Aerobic Exercise

The acute aerobic exercise program consisted of 30 min of moderate-intensity exercise on a cycle ergometer (MONARK 894E, made in Sweden). It began with a 5-min warm-up at a resistance of 0.5 kp. Then, the resistance was adjusted to 1 kp and participants were expected to maintain a rating of perceived exertion (RPE) in the range of 13–15 for 20 min. Finally, the resistance was returned to 0.5 kp for a 5-min cool-down. The target heart rate (HR) across moderate-intensity aerobic exercise was 60~70% maximum HR (maximum HR = 220-age), according to the American College of Sports Medicine guidelines. Participants’ HR data were collected via Polar heart rate monitors (RCX3, made in Sweden). HR, ratings of perceived exertion (RPE), and revolutions per minute (RPM) measures were obtained from participants every 3 min during exercise.

#### 2.2.2. DM Task

The DM task consisted of word learning followed by three free recall tests and a recognition test, all of which were administered in Eprime 2.0 (Psychology Software Tools, Pittsburgh, PA, USA). Participants were asked to learn 21 Chinese two-character emotionally neutral words (arousal level of 5.04 ± 0.27 on a 9-point scale) from a pool of 126 words taken from the Emotional Information of Modern Chinese Two-character Word Evaluation Table [21]. These words were randomly assigned to six groups, with 21 words in each group (7 verbs, 7 nouns, and 7 adjectives).

**Figure 1:**
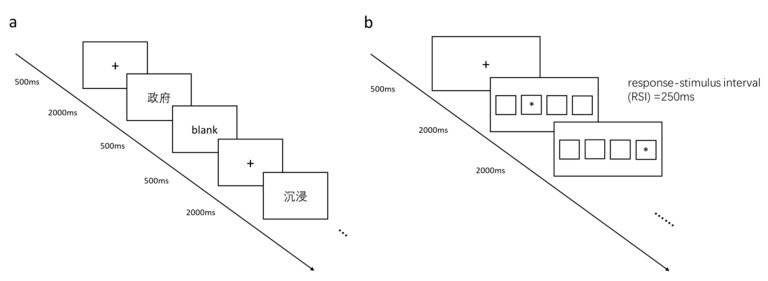
Behavioral task summary. Flow charts of the free recall task (**a**) and serial reaction time task (**b**).

For the word learning phase, first, a black cross was displayed on a white-background computer screen for 500 ms (refresh rate 60 Hz, resolution ratio 1366 × 768). Each learning target word was shown in black (font size 34) at the center of the white screen for 2 s and followed by a 500-ms black screen (Figure 1a). Each participant was presented with 21 Chinese two-character neutral words in a random order without duplication, and participants were asked to memorize as many of the presented words as possible.

After word learning, participants performed free recall tasks, which required them to write down as many target words as they could within 100 s in any order. After completing the first free recall task, which was conducted immediately after word learning, the participants were reminded not to rehearse the words. Participants completed a 1-h free recall task and 24-h free recall task for the same target words. The numbers of words recalled correctly were recorded for each recall trial.

The recognition test was taken after the last free recall task. It included the 21 target words intermixed randomly with 21 additional distracting words, with no duplications. Participants were asked to press designated buttons (counter-balanced across subjects) to indicate whether they thought each word on the screen had been in the word learning exercise or not.

#### 2.2.3. PM Task

A SRTT was used to assess PM in Eprime 2.0. As shown in Figure 1b, the SRTT began with a black cross being displayed on a white screen for 500 ms. Then, four squares (4 cm × 4 cm) were situated in equidistant positions along a central horizontal line on a white screen. The target stimulus was a black asterisk (“*”; font size 34) at the center of one of aforementioned squares in a set sequence. Participants were asked to press the appropriate keyboard key (d, f, j, or k) designating the position of the target stimulus from left to right in the array. The d, f, j, and k keys were pressed with the left middle finger, left index finger, right index finger, and right middle finger, respectively. The target stimulus was shown for 2000 ms followed by a 250-ms response-to-stimulus interval.

Three 12-stimulus sequences were utilized in the SRTT, which were taken from previous studies [22,23]: sequence 1, 1-2-4-3-1-3-2-1-4-2-3-4; sequence 2, 4-3-2-4-2-3-1-2-1-4-1-3; and sequence 3, 3-4-3-2-1-3-1-4-2-4-1-2. The numbers 1, 2, 3, and 4 represented the four positions on the screen where the target stimuli could appear, from left to right. The SRTT consisted of a sequence learning stage, a 1-h test, and a 24-h test. Ten practice stimuli were delivered prior to the formal experiment. The learning stage was comprised of eight blocks with a 10-s inter-block rest interval. In each block, 76 stimuli were presented. In the first six blocks and the last block, the first 4 stimuli were ordered randomly, and the remaining 72 stimuli included six iterations of one of the above predetermined 12-stimulus sequences. In the seventh block, all 76 stimuli were presented in a random order.

Prior to the experiment, participants were told that the purpose of the PM experiment was to examine reaction time and that they should respond to target stimuli by pressing the corresponding keys as quickly and accurately as possible. After completing all PM experiments, participants were asked if they observed any particular regularity to probe whether selective implicit learning had been achieved.

### 2.3. Procedure

**Figure 2:**
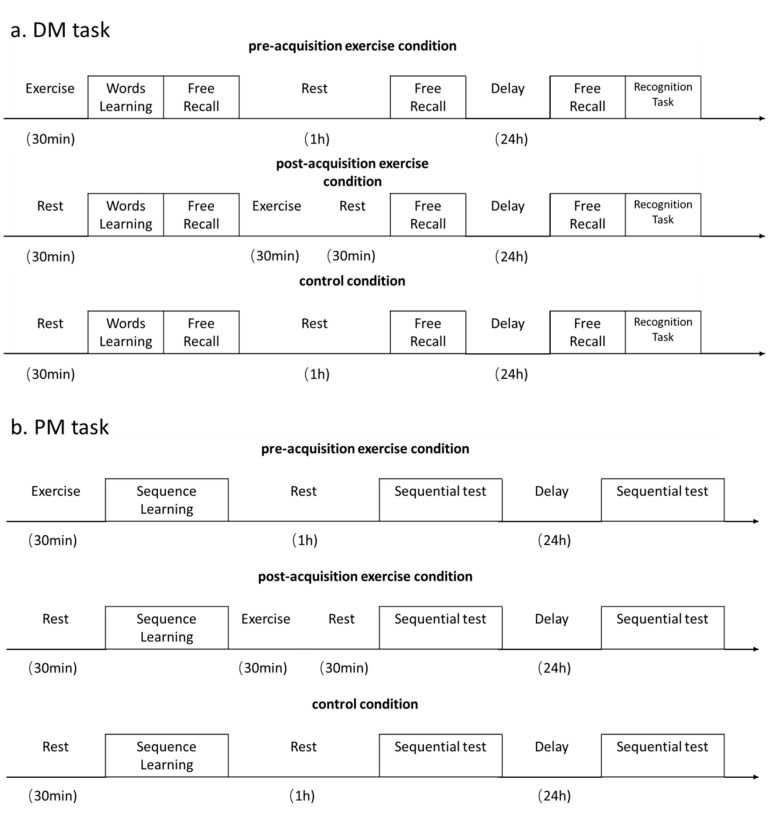
Task flow and experimental conditions for the declarative memory (DM) (**a**) and procedural memory (PM) (**b**) tasks.

As summarized in Figure 2, all participants completed the DM task and PM task under three conditions: pre-acquisition exercise, post-acquisition exercise, and no exercise (control). Specifically, in the pre-acquisition exercise condition, participants completed the exercise program before the entire memory task such that the exercise could affect encoding and early consolidation. In the post-acquisition exercise condition, participants completed the exercise program after the first free call task (DM experiment) or sequence learning stage (PM experiment) such that the exercise could influence only consolidation. In the control condition, participants had a rest or read a magazine instead of exercising. These three conditions were ordered randomly in a balanced manner and carried out 1 week apart. Half of the participants first completed the DM task (all three experimental conditions), and the other half first completed the PM task. Participants were asked to not consume caffeine or alcohol and to not engage in strenuous exercise within a 2 h period before the experiment.

Take the DM task (a) as an example. In the pre-acquisition exercise condition, each participant completed a 30-min exercise program and then started the word learning section when his HR had returned to 110% of his resting HR. Immediately after the word learning stage, participants completed the first free recall test and then completed the second free recall test after a 1-h rest. Participants completed the third free recall test and the recognition test 24 h after the learning stage. The protocol for the post-acquisition exercise condition differed from the pre-acquisition exercise condition only in that participants rested prior to word learning and the first free recall test and then completed the same 30-min exercise program exactly as in the pre-acquisition exercise condition, except that it was completed immediately following the first recall test. They then had a 30-min rest prior to completing the 1-h second recall test. In the post-acquisition exercise condition, participants completed the 24-h recall test and recognition test in succession 24 h after the learning stage. In the control condition, participants completed the same learning stage and tests as in the other two conditions but were not engaged in an exercise program at any time.

### 2.4. Data Analysis

#### 2.4.1. Behavioral Data Processing

For the PM task, reaction time and accuracy rate were analyzed. As per previous work [24,25], trials containing errors (i.e., an incorrect keystroke response by the subject) and reaction times greater than 1200 ms or less than 200 ms were discarded. For the DM task, the numbers of words recalled correctly in the three free recall tasks and the numbers of correct (e.g., responding ‘‘learned’’ when a learned word was actually present) and incorrect responses (e.g., responding “learned’’ to a distracting word) in the recognition test were analyzed. The recognition rate was calculated from the difference between separately computed hit rates and false alarm rates. Discrimination index (d′) and response bias (*β*) values were calculated according to signal detection theory [26].

Data from three participants were discarded due to outlier data falling outside three standard deviations around the mean. Data from one more participant was removed because he reported recognizing the repeating sequence pattern after completing the PM task. Therefore, data from 19 and 18 participants were considered valid for the DM task and PM task, respectively, and thus incorporated in the final statistical analysis.

#### 2.4.2. Statistical Analysis

One-way analyses of variance (ANOVAs) and repeated measures ANOVAs were used to analyze the data in SPSS 23.0 software (SPSS Inc., Chicago, IL, USA) with the Greenhouse Geisser adjustment. The least significant difference method was used for further comparisons. Effect size was represented by partial *η*^2^ values. Mean values for behavioral variables are reported with standard deviations. For all statistical analyses, the alpha significance level was set at 0.05.

To be specific, differences in recall performance across free recall tests conducted at different post-learning delays (immediate, 1 h, 24 h) and under different experimental conditions (pre-acquisition, post-acquisition, control) were assessed with a two-way (time × condition) repeated measures ANOVA. For the recognition test, a one-way repeated measures ANOVA was conducted to compare differences among the three exercise conditions. For the SRTT, differences between exercise conditions and task stages were analyzed with a two-way (stage × condition) repeated measures ANOVA. Additionally, to detect effects of exercise condition and learning sequence within each of two SRTT test time points, two separate two-way 3 (blocks 6–8) × 3 (experimental conditions) repeated measures ANOVAs were employed.

## 3. Results

### 3.1. HR, RPE and RPM during Exercise

**Figure 3:**
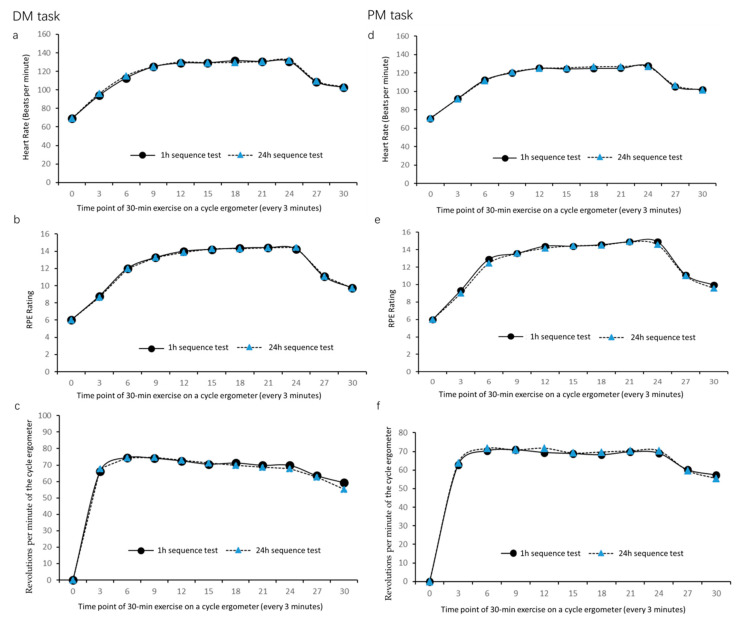
Physiological data obtained during the DM task and PM task. (**a**–**c**) DM task: heart rate (HR) (**a**); rating of perceived exertion (RPE) (**b**); and revolutions per minute (RPM) (**c**). (**d**–**f**) PM task: HR (**d**); RPE (**e**); and RPM (**f**). For both tasks, data were collected every 3 min during the exercise bout.

A two-way 2 (exercise condition: pre- and post-acquisition exercise) × 11 (point-in-time of record) repeated measures ANOVA was applied to analyze HR, RPE, and RPM data from the DM task, and a similarly structured ANOVA was applied to analyze the same variables from the PM task (Table 1). We found that that HR, RPE, and RPM did not differ significantly between the two exercise conditions in either the DM task or the PM task (all *p*s > 0.05; Figure 3).

### 3.2. DM

#### 3.2.1. Free Recall Tests

**Figure 4:**
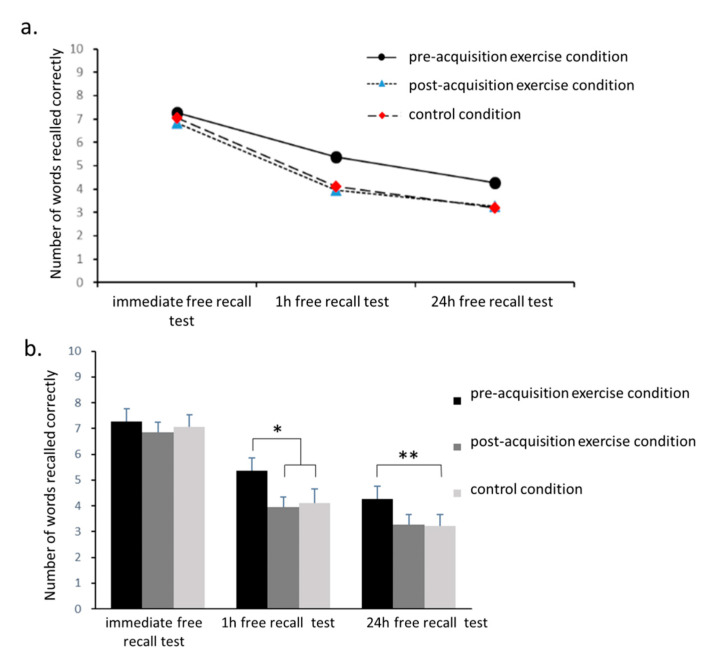
Comparisons of the number of words recalled correctly in the PM experiment. (**a**) Comparisons over time. (**b**) Comparisons between conditions. * *p* < 0.05, ** *p* < 0.01.

A two-way 3 (point-in-time of recall) × 3 (experimental conditions) repeated measures ANOVA of the number of words correctly recalled revealed a significant main effect of time (*F*_2,36_ = 112.8, *p* < 0.001, *η*_p_^2^ = 0.9). As shown in Figure 4a, recall performance showed a significant decreasing trend with increased time between learning and recall (immediate test, 7.1 ± 2.0 words; 1 h test, 4.5 ± 2.2 words; and 24 h test, 3.6 ± 2.0 words). In addition, there was a significant interaction between experimental condition and time (*F*_4,72_ = 3.3, *p* = 0.03, *η*_p_^2^ = 0.16). As shown in Figure 4b, the number of correctly recalled words was significantly greater in the pre-acquisition exercise condition (5.4 ± 2.1 words) than in the other two conditions (control, 4.1 ± 2.4 words, *p* = 0.01; post-acquisition exercise, 3.9 ± 1.7 words, *p* = 0.01). Furthermore, in 24 h free recall test, the number of words correctly recalled in the pre-acquisition exercise condition (4.3 ± 2.1 words) was significantly greater than in the control condition (3.2 ± 2.0 words, *p* = 0.005).

#### 3.2.2. Recognition Test

**Figure 5:**
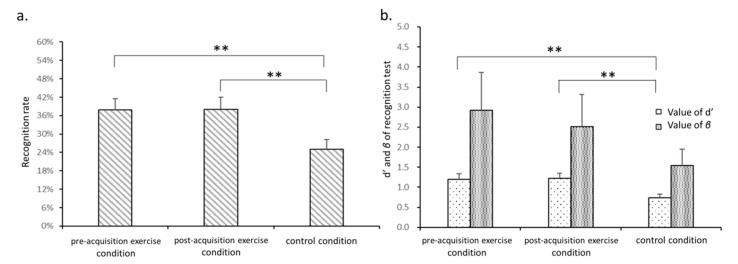
Comparison of word recognition rate (**a**) and of d’ and *β* values (**b**) in the DM experiment across experimental conditions; ** *p* < 0.01.

A one-way repeated measures ANOVA of recognition rate across the three experimental conditions revealed a significant main effect of condition on recognition rate (*F*_2,36_ = 7.0, *p* = 0.003, *η*_p_^2^ = 0.3). As shown in Figure 5a, recognition rate in the control condition (25.1% ± 3.1%) was significantly lower than in pre-acquisition exercise (37.9% ± 3.6%, *p* = 0.002) and post-acquisition exercise (38.1% ± 3.9%, *p* = 0.007) conditions, demonstrating that recognition was better in both exercise conditions than in the control condition.

Meanwhile, a one-way repeated measures ANOVA of d’ also revealed a significant main effect of condition (*F*_2,36_ = 8.6, *p* = 0.001, *η*_p_^2^ = 0.3). As shown in Figure 5b, d’ was significantly greater in the pre-acquisition exercise (1.2 ± 0.6, *p* = 0.002) and post-acquisition exercise (1.2 ± 0.6, *p* = 0.002) conditions than in the control condition (0.7 ± 0.4). No significant main effect of condition on *β* was found (*p* > 0.05), indicating that the tendency to respond was independent of the experimental condition.

### 3.3. PM Task

**Figure 6:**
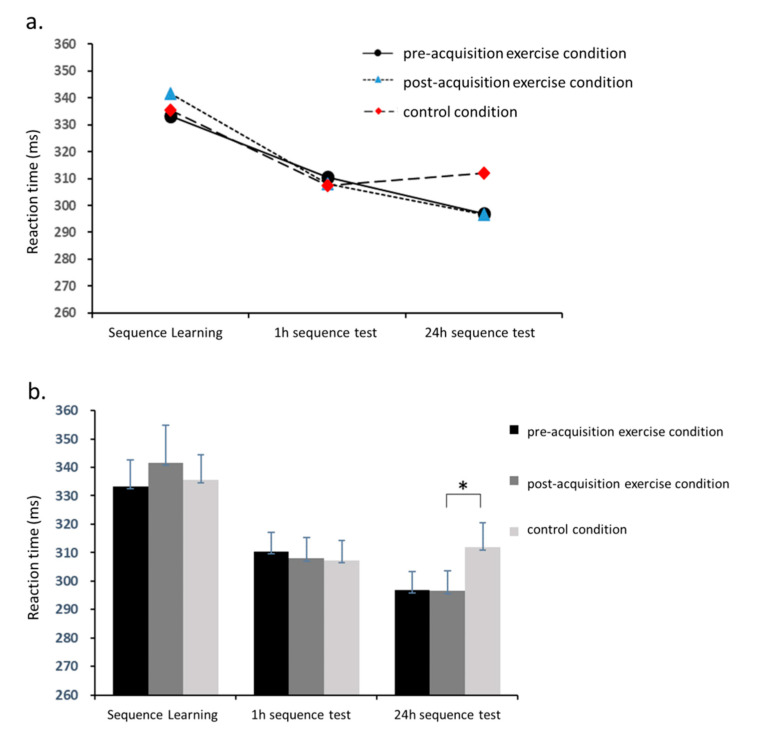
Comparison of reaction times in the serial reaction time task in the PM experiment. (**a**) Comparisons over time. (**b**) Comparisons between conditions. * *p* < 0.05.

A two-way 3 (experimental condition) × 3 (stage: sequence learning, 1 h sequence test, and 24 h sequence test) repeated measures ANOVA revealed a main effect of stage on reaction time (*F*_2,34_ = 62.3, *p* < 0.001, *η*_p_^2^ = 0.8) in the SRTT. As shown in Figure 6a, there were significant progressive improvements in reaction time across all three stages, from the sequence learning stage (336.8 ± 44.1 ms) to the 1 h sequence test (308.6 ± 29.1 ms) and to the 24 h sequence test (301.8 ± 31.7 ms). In addition, there was a significance interaction between experimental condition and task stage (*F*_4,68_ =2.7, *p* = 0.04, *η*_p_^2^ = 0.1). Further analysis revealed (Figure 6b) that mean reaction time in the second sequence test was significantly shorter in the post-acquisition exercise condition (296.6 ± 29.9 ms) than in the control condition (311.9 ± 36.5 ms, *p* = 0.04).

Accuracy rates did not differ significantly among the three conditions, indicating that there was not an exchange effect of either accuracy or speed between participants’ response times and accuracy rates. Thus the analysis based on reaction time can be considered fairly reliable.

**Figure 7:**
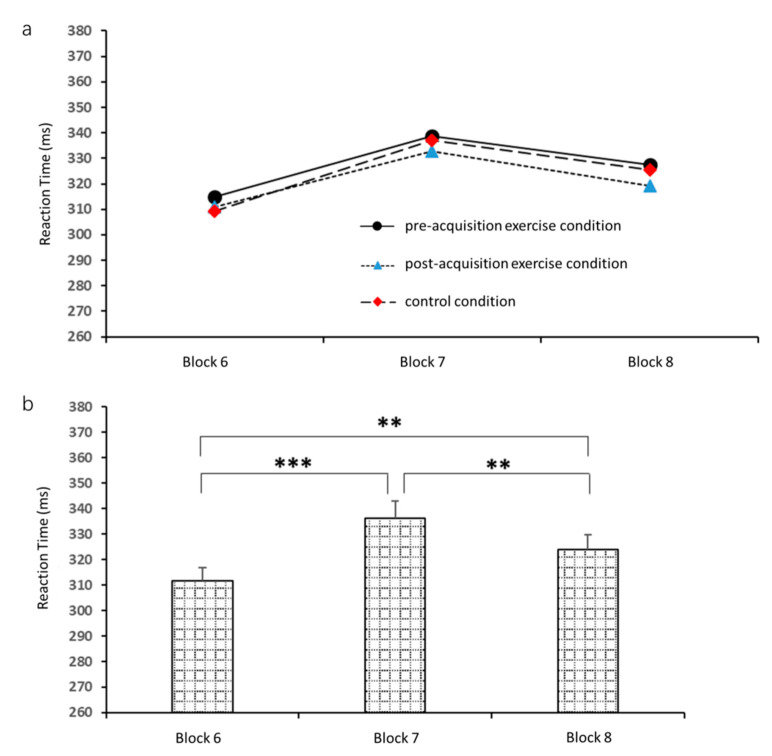
Mean reaction times in the 1-h sequence test by block and by exercise condition. (**a**) Comparison of reaction times among blocks 6, 7, and 8. (**b**) Comparison of reaction times among pre-acquisition exercise, post-acquisition exercise, and control conditions. ** *p* < 0.01, *** *p* < 0.001.

A two-way 3 (block: 6, 7 and 8) × 3 (experimental condition) repeated measures ANOVA of reaction time in the 1-h sequence test revealed a significant main effect of block (*F*_2,34_ = 16.2, *p* < 0.001, *η*_p_^2^ = 0.5), but not a main effect of exercise condition (*F*_2,34_ = 0.3, *p* = 0.8, *η*_p_^2^ = 0.02); moreover, there was no significant block × exercise condition interaction (*F*_4,68_ = 0.2, *p* = 0.9, *η*_p_^2^ = 0.01). Further analysis revealed (Figure 7) that reaction time was significantly shorter in block 6 (311.7 ± 29.2 ms) than in block 7 (336.2 ± 36.4 ms, *p* < 0.001) and block 8 (324.0 ± 36.9 ms, *p* = 0.004). Additionally, reaction time was significantly shorter in block 8 than in block 7 (*p* = 0.02).

**Figure 8:**
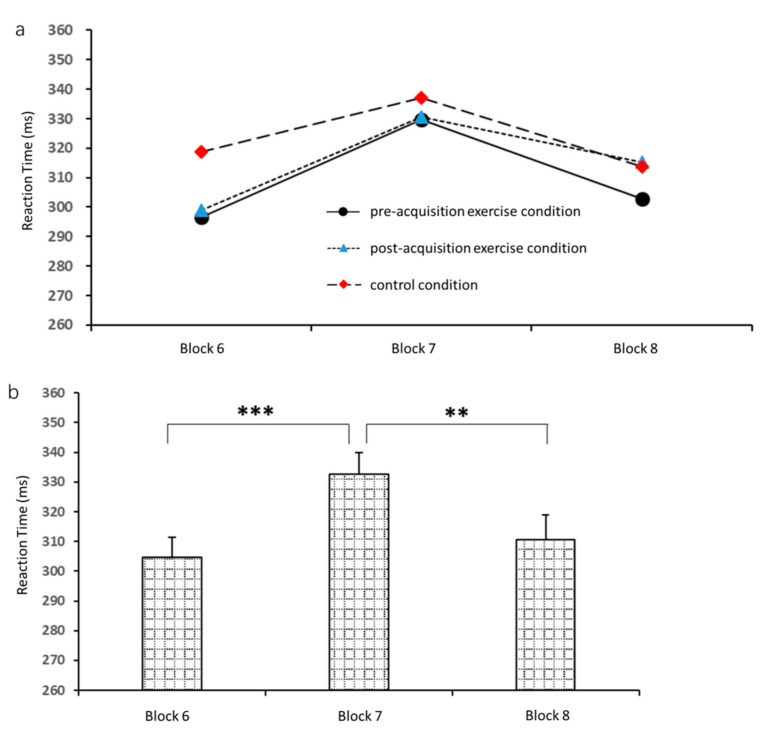
Mean reaction times in the 24-h sequence test by block and by exercise condition. (**a**) Comparison of reaction times among blocks 6, 7, and 8. (**b**) Comparison of reaction times among pre-acquisition exercise, post-acquisition exercise, and control conditions. ** *p* < 0.01, *** *p* < 0.001.

Additionally, a two-way 3 (block) × 3 (exercise condition) repeated measures ANOVA of reaction time in blocks 6, 7, and 8 of the 24-h sequence test revealed a significant main effect of block (*F*_2,34_ = 19.1, *p* < 0.001, *η*_p_^2^ = 0.5), but not of exercise condition (*F*_2,34_ = 2.0, *p* = 0.1, *η*_p_^2^ = 0.1); and there was not a significant block × exercise condition interaction (*F*_2,34_ = 2.4, *p* = 0.06, *η*_p_^2^ = 0.1). Further analysis (Figure 8) revealed that reaction time was significantly shorter in block 6 (304.7 ± 34.3 ms) than in block 7 (332.6 ± 36.6 ms, *p* < 0.001), and reaction time in block 8 (310.6 ± 39.6 ms) was significantly shorter than in block 7 (*p* = 0.001). Reaction times in block 6 and block 8 were statistically similar (*p* = 0.1).

## 4. Discussion

The current study examined the effects of acute aerobic exercise on DM and PM and showed that acute aerobic exercise enhanced intermediate-term and long-term DM and long-term PM memory via influences on different processing periods. Specifically, acute aerobic exercise appeared to improve DM performance mainly through a positive impact on encoding, but appeared to improve PM performance mainly through facilitation of memory processes during the consolidation period.

HR, RPE, and RPM data in the pre- and post-acquisition exercise condition indicated that all subjects performed moderate-intensity aerobic exercise with RPE values in the range of 13–15. All subjects’ HRs remained within the range of 60–70% of maximal HR throughout the exercise session. Furthermore, because exercise intensity levels in the pre- and post- acquisition exercise conditions were similar, differences in free recall, recognition, and SRTT performance cannot be attributed to differences in exercise intensity.

### 4.1. Acute Aerobic Exercise Enhances Primarily DM Encoding

The DM experiment showed that acute aerobic exercise before, but not after, DM learning had significant positive effects on intermediate-term (1 h) and long-term (24 h) recall test performance. These results are in agreement with previous studies in which subjects showed 1-h and 24-h memory enhancement in response to performing 30 min of moderate intensity acute aerobic exercise prior to memorizing declarative material [4,17]. Exercise completed shortly before the learning stage can potentially affect encoding processes as well as early consolidation processes, and prior studies have reported positive effects of exercise performed after word learning or after the first recall test on long-term memory, which suggests an influence on consolidation [17,27]. However, in this study, we did not obtain significant facilitatory effects of post-acquisition exercise on free recall performance 1 h or 24 h later, suggesting that it was not effective for facilitating long-term memory consolidation.

Contrasting with our free recall test results, our recognition test results showed that either pre- or post-acquisition exercise enhanced word recognition compared to the control (no exercise) condition, which indicates that acute aerobic exercise may enhance both encoding and early consolidation of long-term DM, as has been suggested by previous research. Notably, Hötting et al. examined German-Polish word retention acquired before low- or high-intensity exercise for 30 min and found that only the high-intensity exercise group performed better than the control group in a 24-h recognition test [28]. Etnier et al. also observed an enhancing effect of high-intensity acute aerobic exercise prior to encoding on 24-h recognition test performance [29]. In the present study, subjects’ memory performance benefitted from moderate-intensity exercise, suggesting that at least a moderate intensity of acute aerobic exercise near the time of learning may be needed to improve long-term recognition performance 24 h later. Labban did not observe enhanced 24-h recognition test performance with either pre- or post-acquisition exercise [17]. However, Labban’s recognition test included only 15 learned words and 15 interference words, which may have been too easy and thus not revealed significant effects. In our study, we observed post-acquisition exercise-associated enhancement of 24-h recognition test performance but not of 1-h or 24-h free recall test performance. It may be that moderate-intensity acute aerobic exercise affects DM consolidation processes, but that the effect size is more pronounced with pre-acquisition exercise than with post-acquisition exercise. The absolute numbers of words recalled were greater in the pre-acquisition exercise condition than in the post-acquisition exercise condition, albeit not significantly so. Importantly, a recognition test, in which one is required only to identify target words, is easier than a free recall test, in which one needs to produce the words, making the recognition test more sensitive to mild enhancing effects.

Altogether, our DM experimental results suggest that acute aerobic exercise may have a pronounced enhancing effect on encoding as well as potentially a mildly enhancing effect on early consolidation of DM. These results thus partially support our hypothesis that acute aerobic exercise enhances the DM encoding processes.

### 4.2. Acute Aerobic Exercise Enhances Primarily PM Consolidation

Our SRTT results indicated that moderate-intensity acute aerobic exercise enhanced implicit sequence learning performance 24 h after the learning phase. Notably, exercise had a stronger promoting effect when performed after, as opposed to before, the sequential learning phase. Thus, contrasting with our DM experiment findings, an exercise bout prior to the consolidation period enhanced long-term PM, consistent with our hypothesis.

The present PM experiment results fit with findings from a recent study [18], wherein a high-intensity acute aerobic exercise program performed either before or after learning was shown to enhance motor memory learning. The enhancement effects were not significant relative to non-exercise controls at a 1-h test, but they became significant at a 24-h test, with post-learning exercise having a more pronounced enhancing effect at the 24-h test than pre-learning exercise. Moreover, the better PM performance associated with post-learning exercise remained more pronounced than that associated with pre-learning exercise after 7 days. Although the current study did not include a 7-day memory test, it is noteworthy that the PM enhancement in our study was not apparent after 1 h but rather became significant after a 24-h delay. This difference between our 1-h and 24-h PM results is consistent with previous studies. Ostadan et al. examined acute aerobic exercise effects on retention of serial memory 8 h later and found that subjects who engaged in post-acquisition exercise performed better than controls [30]. Similarly, McNerney and Radvansky indicated that serial reaction times remained enhanced relative to controls 7 d after training in subjects who engaged in either pre- or post-acquisition exercise [31]. Together with these prior studies, our results show that acute aerobic exercise can enhance long-term PM, mainly facilitating PM consolidation processes, with a relatively slow facilitatory time course.

Although we balanced exercise conditions and sequence tasks across subjects, because the second experiment had a within-subjects design, it is possible that the enhancement effect of exercise on PM was influenced by a practice effect. In the 1-h sequence test, reaction times in blocks 6 and 8 were significantly shorter than in block 7 (the random sequence block), but reactions times in block 6 were also shorter than those in block 8. Thus, the pattern of results in the 1-h sequence test is consistent with a possible practice effect. In contrast, in the 24-h sequence test, we observed shorter reaction times in blocks 6 and 8 than in block 7 (the random sequence block), but there was no significant difference between blocks 6 and 8. Therefore, further consolidation of implicit learning was more evident at the 24-h sequence test than at the 1-h test, overtaking any practice effect that may have occurred. This finding further suggests that acute aerobic exercise enhancement of PM performance is attributable mainly to enhancement of consolidation over many hours.

In summary, our PM experiment results indicate that acute aerobic exercise can facilitate PM consolidation processes. These consolidation enhancing effects yielded improved long-term PM performance.

### 4.3. Potential Mechanisms of Distinct Exercise-Induced Effects on Formation of DM and PM

Building upon previous findings, in the present study, we found different effects of acute aerobic exercise on DM and PM. DM is an explicit form of memory that stores information related to facts and events, whereas PM is an implicit form of memory that stores sensorimotor information acquired in motor skill learning [9]. The differential effects of exercise on DM and PM could be related to the fact that these two forms of memory involve different brain areas. DM is highly dependent on superficial temporal lobe structures, especially the hippocampus, whereas PM is dependent on deep subcortical structures, such as the striatum, as well as the cerebellum [32].

The facilitating influence of acute aerobic exercise on DM and PM also differed in relation to time course. Exercise enhanced DM in a manner that was reflected 1 h and 24 h after learning, whereas exercise enhancement of PM became apparent 24 h after learning. It has been hypothesized that acute aerobic exercise effects on cognitive functions may be attributed to exercise induced increases in arousal [33]. If so, exercise induced arousal may facilitate DM encoding processes and PM consolidation processes, thereby improving long-term memory performance. Importantly, some neurochemicals released during acute aerobic exercise, such as brain-derived neurotrophic factor [34], epinephrine [35], and dopamine [36], may respectively facilitating encoding process of DM and consolidation process of PM. Moreover, due to the processing times of encoding and consolidation being inconsistent, the enhancing effect of acute aerobic exercise on DM and PM differed in time as well.

### 4.4. Limitations

Although we used a widely used maximum HR estimation formula recommended by the American College of Sports Medicine, true maximum HRs differ among individuals. Consequently, exercise at the same HR may differ in actual intensity across individuals. In future studies, we can clarify exercise intensity domains by employing a more flexible graded exercise test. Exercise intensity might also affect experimental results to some extent. Some studies have reported enhancing influences of high-intensity acute aerobic exercise on long-term memory. Thus, to obtain a systematic understanding of the complex relationships among intensity of exercise, time, and particular types of memory, it would be of interest to conduct retention tests at time points that represent a longer delay after the intervention and to examine the effects of different intensities of aerobic exercise. Meanwhile, all of our participants were male college students. Further examination of potential effects of gender and age on the effects of acute aerobic exercise on memory processing, which some evidence has pointed to [37,38], should be studied further. Additionally, the current study provides behavioral evidence of the impacts of exercise on memory processing but does not provide information about the neural mechanisms underlying these effects, which should be examined in future studies.

## 5. Conclusions

Acute aerobic exercise can enhance long-term DM and PM. Our data indicate that acute aerobic exercise mainly facilitates the encoding period of DM and the consolidation period of PM.

## Figures and Tables

**Table 1:** Mean HR, RPE, and RPM (±SD) under pre- and post-acquisition exercise conditions.

Variable	Pre-Acquisition Exercise		Post-Acquisition Exercise	
	DM Task	PM Task	DM Task	PM Task
HR, beats per minute	126.9 ± 6.1	122.9 ± 4.8	127.4 ± 5.3	123.2 ± 5.2
RPM	71.8 ± 1.8	69.5 ± 0.8	71.4 ± 2.6	70.6 ± 0.9
RPE	13.8 ± 0.8	13.6 ± 0.7	13.8 ± 0.8	14.1 ± 0.8
Exercise intensity, percent maximum HR	64.0 ± 3.1%	61.6 ± 2.4%	64.3 ± 2.7%	61.8 ± 2.6%

HR, heart rate; RPM, revolutions per minute; RPE, rating of perceived exertion. DM task, *N* = 19; PM task, *N* = 18.
